# Distinct and Shared Determinants of Cardiomyocyte Contractility in Multi-Lineage Competent Ethnically Diverse Human iPSCs

**DOI:** 10.1038/srep37637

**Published:** 2016-12-05

**Authors:** Martin L. Tomov, Zachary T. Olmsted, Haluk Dogan, Eda Gongorurler, Maria Tsompana, Hasan H. Otu, Michael Buck, Eun-Ah Chang, Jose Cibelli, Janet L. Paluh

**Affiliations:** 1 State University of New York Polytechnic Institute, Colleges of Nanoscale Science and Engineering (SUNY Poly, CNSE), Nanobioscience, Albany, NY, 12203, USA; 2 University of Nebraska-Lincoln, Electrical and Computer Engineering, Lincoln, NE, 68588, USA; 3 Istanbul University Faculty of Medicine, Institute of Experimental Medicine Research (DETAE), Department of Genetics, Fatih, Istanbul, 34393, Turkey; 4 State University of New York Buffalo (SUNY Buffalo), Department of Biochemistry, Center of Excellence in Bioinformatics, Buffalo, NY, 14203, USA; 5 Eulji Medical Center, Eulji University, Department of Laboratory Medicine, Seoul, Korea; 6 Michigan State University, Departments of Animal Science and Physiology, East Lansing, MI, 48824, USA; 7 LARCEL, Laboratorio Andaluz de Reprogramación Celular, BIONAND, Andalucia, 29590, Spain

## Abstract

The realization of personalized medicine through human induced pluripotent stem cell (iPSC) technology can be advanced by transcriptomics, epigenomics, and bioinformatics that inform on genetic pathways directing tissue development and function. When possible, population diversity should be included in new studies as resources become available. Previously we derived replicate iPSC lines of African American, Hispanic-Latino and Asian self-designated ethnically diverse (ED) origins with normal karyotype, verified teratoma formation, pluripotency biomarkers, and tri-lineage *in vitro* commitment. Here we perform bioinformatics of RNA-Seq and ChIP-seq pluripotency data sets for two replicate Asian and Hispanic-Latino ED-iPSC lines that reveal differences in generation of contractile cardiomyocytes but similar and robust differentiation to multiple neural, pancreatic, and smooth muscle cell types. We identify shared and distinct genes and contributing pathways in the replicate ED-iPSC lines to enhance our ability to understand how reprogramming to iPSC impacts genes and pathways contributing to cardiomyocyte contractility potential.

Human induced pluripotent stem cells (iPSCs; refs 1 and 2) and their differentiated progenitors are providing increased opportunities in personalized medicine, including use in tissue engineering^3^ and in studies to distinguish disease mechanisms from normal development^4^,^5^,^6^. The growing number of potential therapeutic applications of iPSCs include high impact diseases, related to cardiac health^7^ spinal muscular atrophy^8^, neurodegeneration^9^,^10^ and Type I diabetes^11^. Investigation into natural variations based on gender, age and ethnicity and how these contribute to disease and therapeutic outcomes are receiving increased attention that is possible now through inclusion of these parameters in new stem cell resources^12^,^13^,^14^,^15^,^16^. Most challenging to iPSC technology is perhaps ethnic diversity and the additional complexity it brings in distinguishing reprogramming versus ethnic-specific contributions to the generation of multiple functional cells or tissues, which has not yet been investigated. In this study that uses replicate iPSC lines generated from self-designated Asian or Hispanic-Latino apparently healthy donors (Chang *et al*.^13^) we focus on how reprogramming can affect specific cell differentiation pathways. It is not our intention to make broad claims on Asian or Hispanic-Latino ethnicities. Indeed, a future challenge to the stem cell community when expanding ethnically diverse stem cell resources will be in how to compare ethnicities.

Reprogramming remains an inefficient and poorly understood process, in which epigenetic variation and differences in gene expression in iPSC lines reflects an underlying stochastic mechanism^17^. Bioinformatics is playing an increasing role in evaluation of iPSC lines, including development of comparative bioinformatics models that evaluate pluripotency profiles of lines developed under different platforms^18^ and investigation into dynamic changes within lines as cells move from pluripotency through differentiation stages^19^,^20^,^21^. Also critically needed are comparative bioinformatics studies of multiple lines that are derived, differentiated and analyzed under a uniform platform by multiple comprehensive strategies. The ED-iPSC lines analyzed in this study fit this need and include replicate lines of Asian or of Hispanic-Latino designation that were derived from fibroblasts and analyzed under a single platform. In addition, we initiate differentiation protocols from lithography templated uniform EBs to increase accuracy of our comparative analysis^13^,^22^,^23^. In our initial validation of these ED-iPSC cell lines we confirmed normal karyotype, verified pluripotency biomarkers by qRT-PCR, and confirmed teratoma formation as well as *in vitro* tri-lineage commitment, summarized here in Table 1. The current analysis extends these studies substantially to include *in vitro* multi-lineage differentiation to six cell types of high interest for iPSC-derived cell and tissue therapeutic applications along with comparative bioinformatics of replicate lines that revealed differences in generating contractile cardiomyocytes.

In our bioinformatics and cell biological study with replicate Asian (A2.2.2, A2.1.1) and Hispanic-Latino (H3.1.1, H3.3.1) ED-iPSC lines we expand our knowledge of the reprogrammed epigenetic landscape and its impact on generating contractile cardiomyocytes. Stochastic epigenomic differences fall into two primary clusters, each capable of pluripotency as gauged by teratoma and qRT-PCR^13^ as well as our new data on multi-lineage differentiation into pyramidal neurons, CD44+/GFAP+ astrocytes, RPE cells, pancreatic progenitors, smooth muscle and contractile cardiomyocytes. We use previously established protocols for differentiation and initiate differentiation from custom templated uniformly sized EB intermediates for consistency in comparative analysis^22^,^23^. At the epigenetic level, ED-iPSC line reprogramming was evaluated by ChIP-seq for histone modifications H3K4me1 and H3K27ac. The RNA-Seq data of the pluripotent state was used in comprehensive bioinformatics analysis to compare gene expression profiles and identify gene ontology (GO) pathways contributing to observed differences in contractile and non-contractile cardiomyocytes from the Asian and Hispanic-Latino ED-iPSC replicate lines. Contractility was optimized and then quantified in custom microarray wells for ability to generate 3D aggregates, expression of NKX2.5 and GATA4, and expression and banded striations of Troponin-T. By evaluating gene expression in the contractile versus non-contractile ED-iPSC replicate lines, within and across the two replicate pairs, we identify determinants distinct to each of these replicate pairs or shared. We further cross-compare similarly up or down regulated genes as well as inversely regulated genes. Finally, we evaluate implicated GO pathways and discuss those with cardiac relevance, as well as disease associated cardiac pathways. Our findings are expected to be broadly important to the stem cell and biomedical communities by enhancing our understanding of the reprogrammed pluripotent environment and genes, pathways, and alternate regulation that may contribute to in cardiac development.

## Results

### Ectoderm and endoderm multi-lineage differentiation potential of ED-iPSC lines

**Figure 1:**
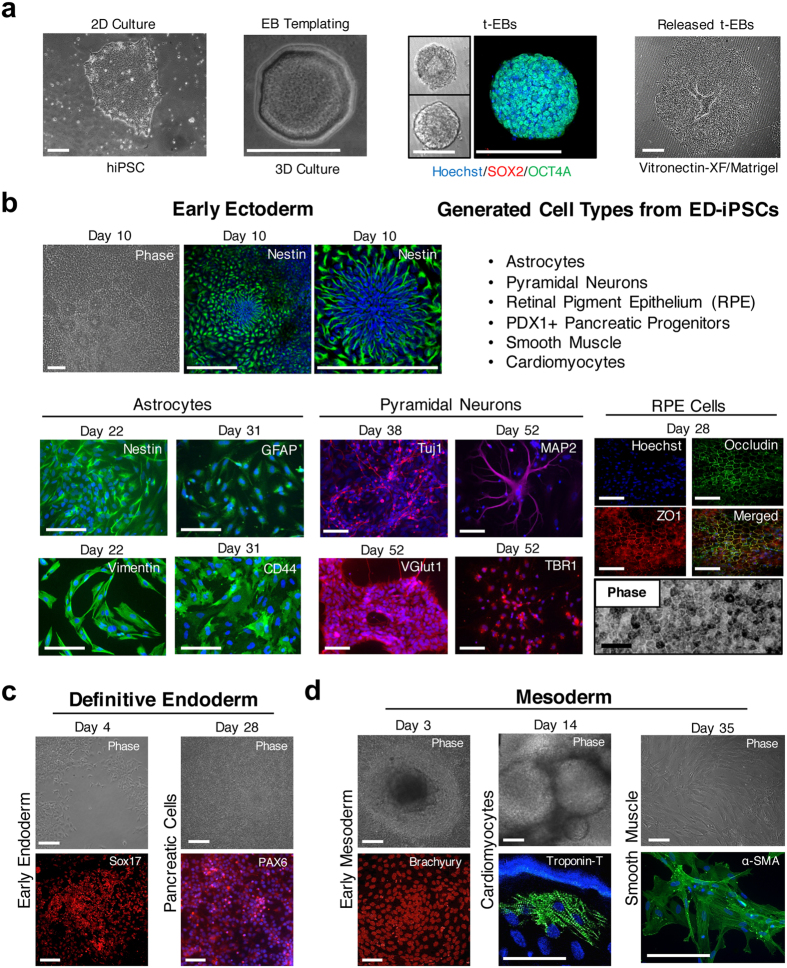
Overview of differentiation strategies applied to ED-iPSC lines. Multi-lineage differentiation to ectoderm, endoderm and mesoderm specialized cell types. (**a**) ED-iPSC lines were maintained as 2D feeder-free cultures, then differentiated from uniformly sized embryoid bodies (EB) formed in custom lithography patterned well arrays (200 μm t-EBs). The t-EBs were plated onto Matrigel or Vitronectin-XF for differentiation. Scale bars are 200 μm. (**b**) Early Ectoderm cell types. Differentiation of plated t-EBs to astrocytes, neurons, and retinal pigment epithelial (RPE) cells was done from neural stem cell (NSC) precursors. Neural rosettes appeared by day 7 upon addition of STEMdiff Neural Induction Medium. Early differentiating neural populations expressed intermediate filament markers Nestin (green; shown ED-iPSC F3.6.1) and Vimentin (green; shown ED-iPSC F3.5.2). Functional astrocytes expressed GFAP (green; shown ED-iPSC H3.3.1) and CD44 (green; shown ED-iPSC A2.2.2) by day 31 of differentiation. Differentiated neurons expressed β-III-tubulin (Tuj1; red; shown is ED-iPSC A2.2.2), MAP2 (red; shown is H9 hESC), VGlut1 (red; shown is ED-iPSC F3.5.2) and TBR1 (red; shown ED-iPSC F3.5.2). Representative differentiation to RPE cells (shown ED-iPSC F3.5.2), stained for Occludin (green) and ZO1 (red) tight junction markers showing cobblestone morphology. Scale bars are 50 μm. (**c**) Definitive endoderm, representative differentiation to pancreatic progenitor cells (shown ED-iPSC A2.2.2). Scale bars are 50 μm. (**d**) Mesoderm cell types. Representative differentiation to cardiomyocytes (shown is ED-iPSC A2.2.2) and smooth muscle cells (shown ED-iPSC F3.5.2). The hESC line WA09 (H9) when compared followed similar protocols. Scale bars are 50 μm.

**Figure 2:**
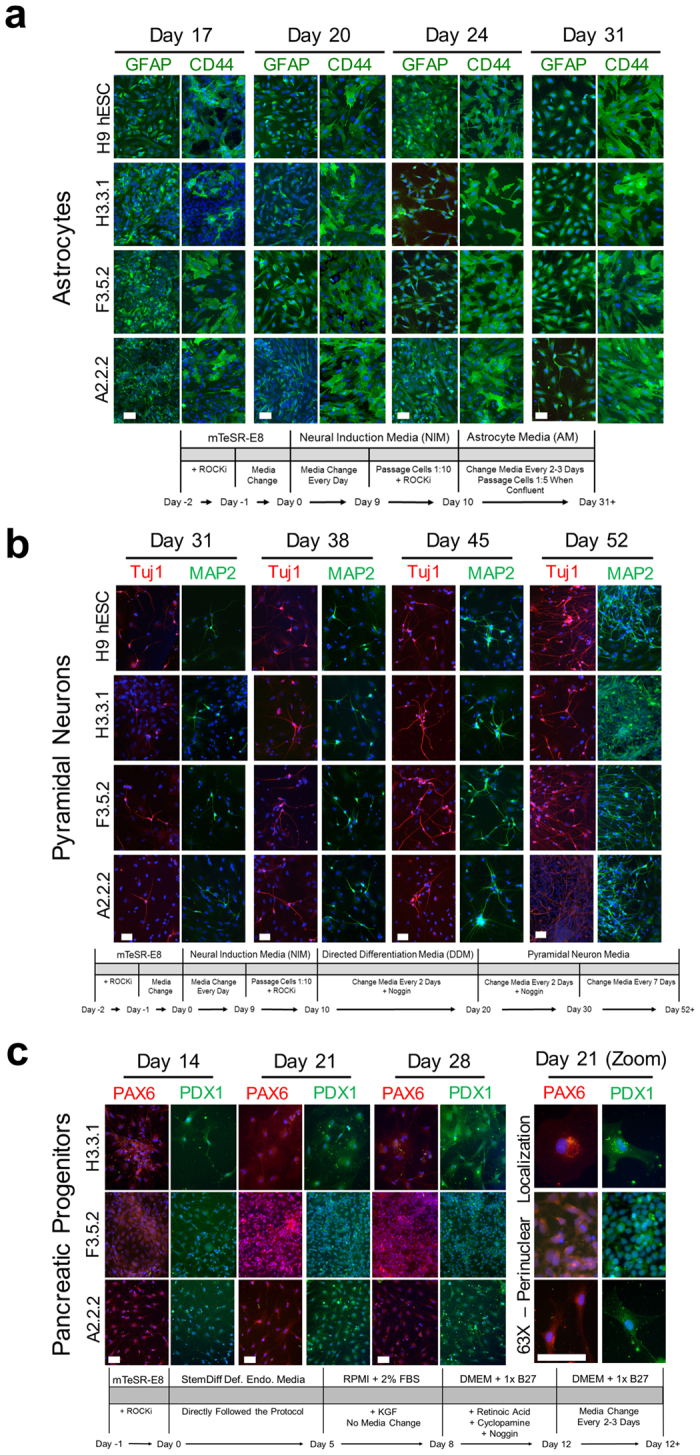
Multi-lineage differentiation of ED-iPSC lines to astrocytes, pyramidal neurons and pancreatic progenitors. Comparative arrays of ED-iPSC lines A2.2.2, F3.5.2 and H3.3.1 showing representative images of differentiation to astrocytes, pyramidal neurons, and pancreatic progenitors following protocols outlined in the text, with detailed differentiation protocol schematics under each corresponding image sets. The hESC H9 control line was also compared for astrocytes and pyramidal neurons. Time frames shown all initiate from the pluripotent stage. (**a**) Astrocyte differentiation. Astrocyte-specific glial fibrillary acidic protein (GFAP) and surface marker CD44 are shown in images acquired at days 17, 20, 24 and 31 of differentiation. (**b**) Pyramidal neuron differentiation. Neuron biomarkers are β-III-tubulin (Tuj1, red) and microtubule associated protein 2 (MAP2, green) are shown in images acquired at days 31, 38, 45 and 52 of differentiation. (**c**) Early pancreatic progenitors. Expression of pancreatic-specific markers PAX6 and PDX1 are shown in images acquired at 14, 21 and 28 days of differentiation, along with a higher magnification image of PAX6 and PDX1 (day 21). Scale bars are 50 μm.

To extend our previous teratoma analysis, biomarker profiles and *in vitro* lineage commitment studies, we used established protocols to generate pyramidal neurons, astrocytes, and retinal pigment epithelium (RPE) of ectoderm origin, as well as pancreatic progenitors arising from endoderm differentiation (Figs 1 and 2). Cell types were chosen based on broad clinical interest in stem cell-derived cell-based therapies for neurodegeneration, injured brain and spinal cord, photoreceptor regeneration with RPE, and diabetes^24^,^25^,^26^,^27^,^28^,^29^,^30^,^31^. All derivations used templated embryoid body (EB) intermediates of uniform size and dimensions (t-EB; Fig. 1a; refs 22 and 23). Neural stem cell (NSC) progenitors were generated essentially as described (Neural Induction Media; StemCell Technologies, Vancouver, Canada). Typical rosette morphology was identified after 7 days of differentiation using brightfield microscopy, and the presence of Nestin in the rosette cells was confirmed by immunocytology (ICC; Fig. 1b). The NSCs were then used to derive astrocytes, and pyramidal neurons. Astrocytes were generated from NSCs by commercial media (Astrocyte Media; Sciencell Research Laboratories, Carlsbad, CA). Positive expression of CD44, GFAP, and Vimentin were monitored in maturing astrocytes (Figs 1b and 2a). In immature dividing astrocytes Nestin and A2B5 were present but decrease over time and plateau with maturation as expected. ED-iPSC lines behaved similarly during astrocyte differentiation (N > 3 replicates). The implemented astrocyte protocol produces primarily CD44+/GFAP+ astrocytes.

Directed differentiation of pyramidal neurons from NSCs was done as previously described (refs 24, 25, 26; Figs 1b and 2b). Time-course and ICC revealed no differences in neuronal differentiation efficiency amongst ED-iPSC lines or when compared to hESC line WA09 (WiCell, Madison, WI) over multiple rounds (N > 3; Fig. 2b). The neural specific biomarker Tuj1 (β-III-tubulin) and neuronal specific MAP2, both present in mature neurons, were used to track the progress of differentiation along with two pyramidal specific biomarkers TBR1 and VGlut1 (Fig. 1b). During differentiation, the Tuj1 and MAP2 biomarkers appeared along with typical neuronal morphology of dendrites and elongated axons as expected. By ICC, 70–80% of the differentiated populations of ED-iPSCs were pyramidal neurons as revealed by specific biomarkers. A representative comparative ICC time-course of differentiation to pyramidal neurons for African-American, Hispanic-Latino and Asian ED-iPSC lines is shown (Fig. 2b). Differentiation into RPE cells from pluripotent t-EBs used established protocols (refs 27 and 32,33,34; Fig. 1b) with no differences observed among the ED-iPSC lines.

Differentiation of ED-iPSCs to pancreatic PDX1+/PAX6+ progenitors, which can generate β-islet cells to address pancreatic diseases, was done using established protocols^35^,^36^,^37^. ICC was used to track PAX6, an early progenitor marker, essential for later development of the endocrine pancreas and specification of hormone production^35^,^38^,^39^, and the biomarker PDX1, which is important in pancreatic development, specifically in α-cells and β-islet cells lineage formation^35^,^36^,^37^,^38^,^40^ (Figs 1c and 2c). PAX6 localizes specifically to the perinuclear region consistent with maturing pancreatic cells, while the co-expressed PDX1 is localized in the nucleus^35^ (Fig. 2c, second panel). Expression of both markers has been reported to be critical for proper pancreatic cell differentiation^40^,^41^. Similar staining was present across ED-iPSC lines and was comparable to differentiated hESC WA09 cells. In addition to the representative three lines shown, differentiation efficacy of ED-iPSC lines F3.6.1, A2.1.1 and H3.1.1 for astrocytes, pyramidal neurons, and pancreatic progenitors revealed no observable differences. RPE differentiation was tested on the three ED-iPSC lines shown in Fig. 2 (A2.2.2, F3.5.2, and H3.3.1). Our studies show no apparent differences between ED-iPSC lines examined and for differentiation towards evaluated ectodermal or endodermal cells.

### ED-iPSC lines similarly generate smooth muscle but differ in ability to generate contractile cardiomyocytes

**Figure 3:**
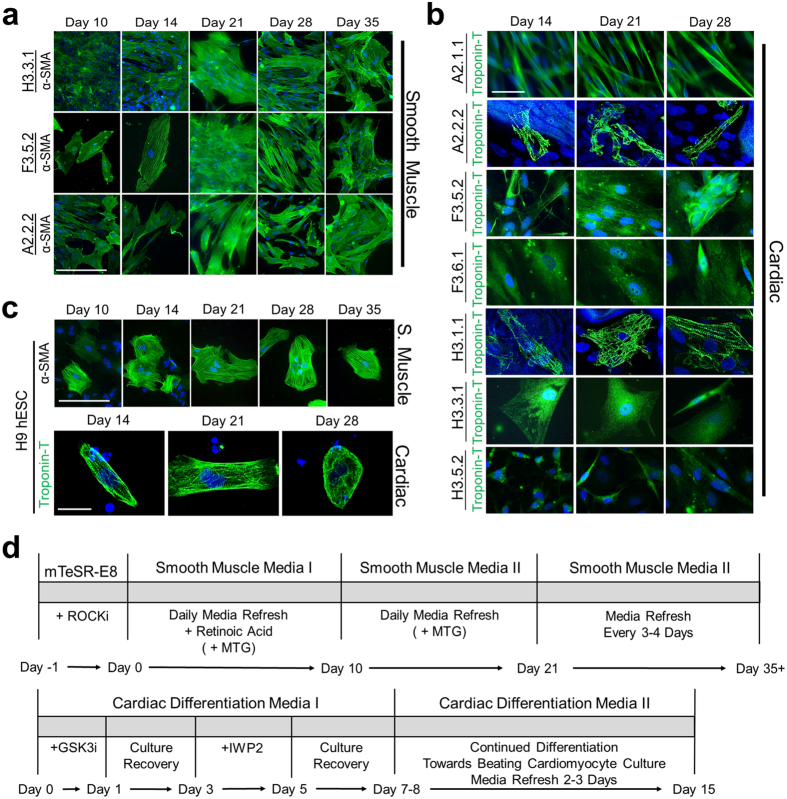
Mesoderm lineage differentiation of ED-iPSC lines to smooth muscle and cardiomyocytes. Comparative arrays of ED-iPSC lines A2.2.2, F3.5.2 and H3.3.1 showing representative images of differentiation to smooth muscle and cardiomyocytes, and compared to hESC H9 following protocols outlined in the text. Time frames shown all initiate from the pluripotent stage. Representative images are shown. (**a**) Smooth muscle. Cells are stained for Hoechst (blue), alpha smooth muscle actin (α-SMA, green). Acquired images show days 10, 14, 21, 28, and 35 of differentiation. (**b**) Cardiomyocytes. Expression of the cardiomyocyte-specific marker Troponin-T (green) is shown at days 14, 21, and 28. ED-iPSC lines with unpolymerized Troponin-T did not develop a contractile phenotype. (**c**) Differentiation of the H9 hESC pluripotent line into smooth muscle and cardiomyocytes. (**d**) Detailed differentiation protocol schematics, top one illustrates smooth muscle differentiation, while bottom one shows cardiomyocyte generation protocol. Scale bars are 50 μm.

Multiple cell types of clinical interest arise from mesoderm including cardiomyocytes, smooth muscle, and cells of hematopoietic lineage amongst others. We examined differentiation to smooth muscle and cardiomyocytes from mesoderm committed Brachyury-T positive lines A2.2.2, F3.5.2, and H3.3.1 (ref. 13; Figs 1d and 3a,b) using established protocols (refs 42, 43, 44, 45; Sciencell Research Laboratories, Carlsbad, CA) and compared to hESC line WA09 (H9, Fig. 3c). All ED-iPSC lines tested readily generated smooth muscle (Fig. 3a). In contrast, the ability to generate 3D aggregated cardiomyocytes with banded Troponin-T, required for contractility, differed in the ED-iPSC lines (Fig. 3b). We observed functional differences within replicate line sets for ED-iPSC Asian lines (A2.1.1 non-contractile versus A2.2.2-contractile) and Hispanic-Latino lines (H3.1.1 contractile versus H3.3.1 non-contractile). The two African American ED-iPSC replicate lines examined revealed weak assembly of Troponin-T (F3.5.2) or diffuse non-banded Troponin-T (F3.6.1) in cardiomyocytes that did not contract. Cardiomyocyte maturation was followed over a time course of 28 days. ICC staining for the cardiac specific Troponin-T biomarker was generally found in regions of the 2D culture that have spontaneously assembled 3D cell clusters. Troponin-T in such 3D aggregated, contractile cardiomyocytes has typical banded striations, as observed in ED-iPSC lines A2.2.2 and H3.1.1 as well as hESC WA09 (H9). We also observed an intermediate Troponin-T phenotype (F3.5.2) and non-banded/diffuse Troponin-T (A2.1.1, F3.6.1, H3.3.1, H3.5.2), each with no contractility. In summary, the ED-iPSC lines, though behaving similarly in smooth muscle differentiation as well as ectoderm and endoderm differentiation, revealed striking differences in generating contractile cardiomyocytes (Fig. 3b, N = 3) that was associated with an inability to assemble 3D clusters with banded Troponin-T.

### Levels of GSK3 inhibition and 3D assembly affect cardiomyocyte generation in ED-iPSCs

**Figure 4:**
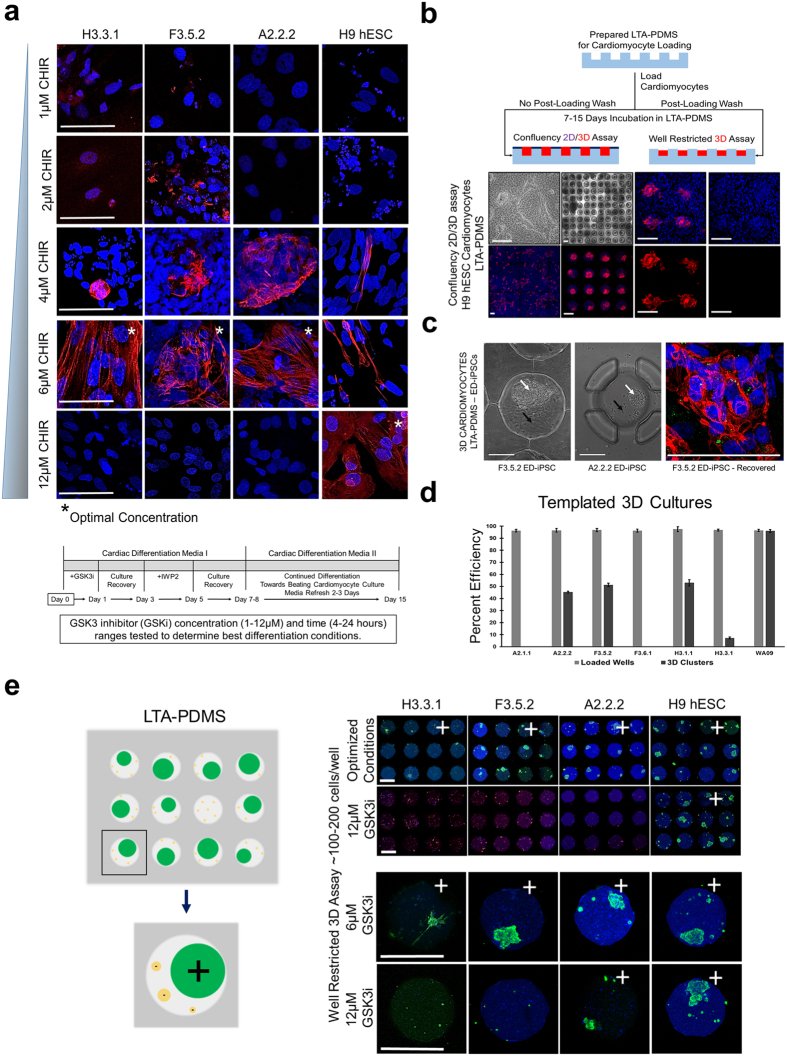
Chemical and physical optimization of ED-iPSC cardiomyocyte protocol. Optimization formation of banded Troponin-T in differentiated ED-iPSC cardiomyocytes was done in in 2D cultures along with ability to form 3D aggregates using custom lithography microwells. Representative images are shown. (**a**) Optimizing cardiac differentiation of ED-iPSC lines A2.2.2, F3.5.2, and H3.3.1, and compared to the H9 hESC line. The GSK3 inhibitor (CHIR99021) optimal concentration and exposure time were individually determined for all lines. Asterisk (*) denotes the optimal concentration of CHIR99021 exposure at 6 hours for each line. Detailed differentiation protocol schematic is shown under the image set. Scale bars are 50 μm. (**b**) Lithography Templated Arrays-PDMS (LTA-PDMS) were used to efficiently quantify 3D aggregation and contractility of cardiomyocytes with Troponin-T positive staining of the same clusters. Last two columns are a zoom of the confluency 3D assays showing a merged image, nuclear stain, Troponin-T and Oct4A negative control. (**c**) First two images are of ED-iPSC clusters patterned in LTA-PDMS wells. White arrows point to 3D clusters, while black arrows mark empty space within the microwell. The third image shows a recovered 3D cardiomyocyte cluster from the F3.5.3 ED-iPSC line and stained for Hoechst (blue) and Troponin-T (red) with characteristic banded morphology typical of contractile cardiomyocytes. Scale bars are 100 μm. (**d**) Histogram of loaded LTA-PDMS grid wells versus generated contractile 3D cardiomyocyte clusters in representative ED-iPSC lines and the control H9 hESC line at optimized differentiation conditions. (**e**) Derived ED-iPSC and H9 hESC cardiomyocytes by our optimized protocol retain banded Troponin-T expression and contractile phenotype when seeded and grown within patterned microarray wells (LTA-PDMS). Scale bars are 200 μm.

Cardiomyocyte differentiation protocols can be optimized by varying GSK3 or IWP2 inhibitor concentrations, as well as time of exposure to these inhibitors^42^,^43^,^46^. Optimization of GSK3 inhibition specifically has been demonstrated to assist Troponin-T banding and contractility. The differentiation protocol was optimized for each non-contractile line (Fig. 4a) by systematically reducing the concentration of the GSK3 inhibitor CHIR99021 (Tocris, Bristol, UK) and time of exposure to 6 hours, from conditions optimal for hESCs (12 μM CHIR, 24–25 hrs) that were used in Fig. 3b. The optimization was done in 2D cultures and then quantified in our custom 3D templated 100 well microarrays (Fig. 4, Supplementary Movies 1–3).

**Figure 5:**
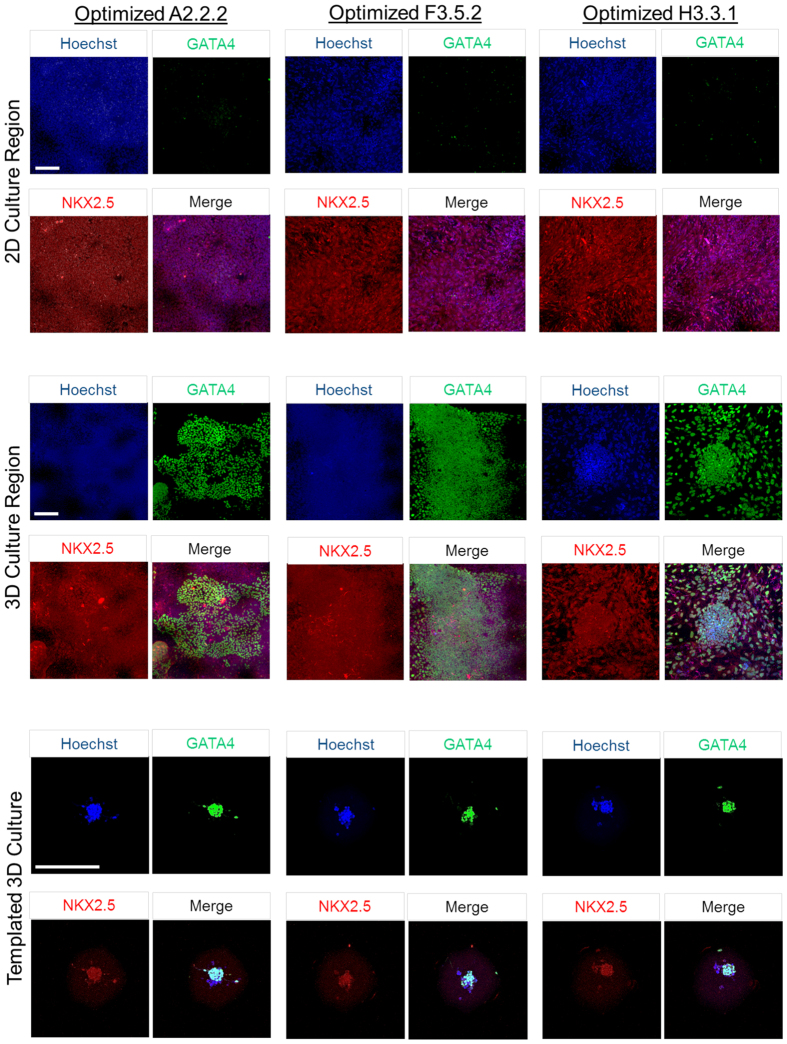
Cardiac biomarker expression of GATA4 and NKX2.5 in ED-iPSC derived cardiomyocytes in 2D and 3D cultures. Expression of early cardiogenic biomarker expression was evaluated in 2D and 3D culture, including templated 3D. Representative ED-iPSC lines A2.2.2, F3.5.2, and H3.3.1 stained for the cardiac specific markers NKX2.5 (red), GATA4 (green) and the Hoechst nuclear stain (blue) are shown under mixed 2D and 3D culture conditions or templated 3D culture in custom lithography generated PDMS microwells. Scale bars are 200 μm.

In order to reproducibly quantify percentage of contractile cardiomyocytes in our protocol optimization, concurrent with 3D aggregation and Troponin-T banding, we dissociated optimized cardiomyocyte 2D cell cultures into single cell suspensions and seeded custom microarray wells at high to low density (Fig. 4b and c, Materials and Methods). The microarray cultures were maintained for one week and 3D self-assembly and development of a contractile phenotype was monitored by phase microscopy and followed by ICC confirmation of cardiac biomarkers (Fig. 4b and c, Fig. 5).

**Figure 6:**
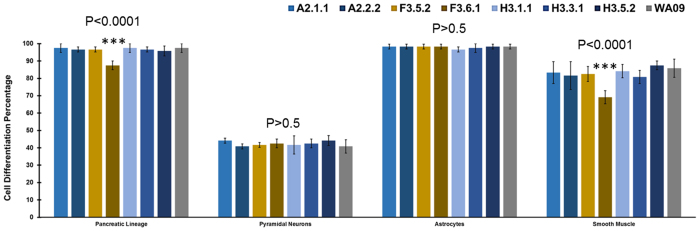
Comparative ED-iPSC differentiation efficiencies to pancreatic progenitors, pyramidal neurons, astrocytes and smooth muscle cells. Histogram summarizing the comparative differentiation efficiency of each experimentally evaluated ED-iPSC line. Statistically significant variability between lines is marked with (***). N > 3 experiments, and 200 cells were counted in total per differentiation condition.

**Figure 7:**
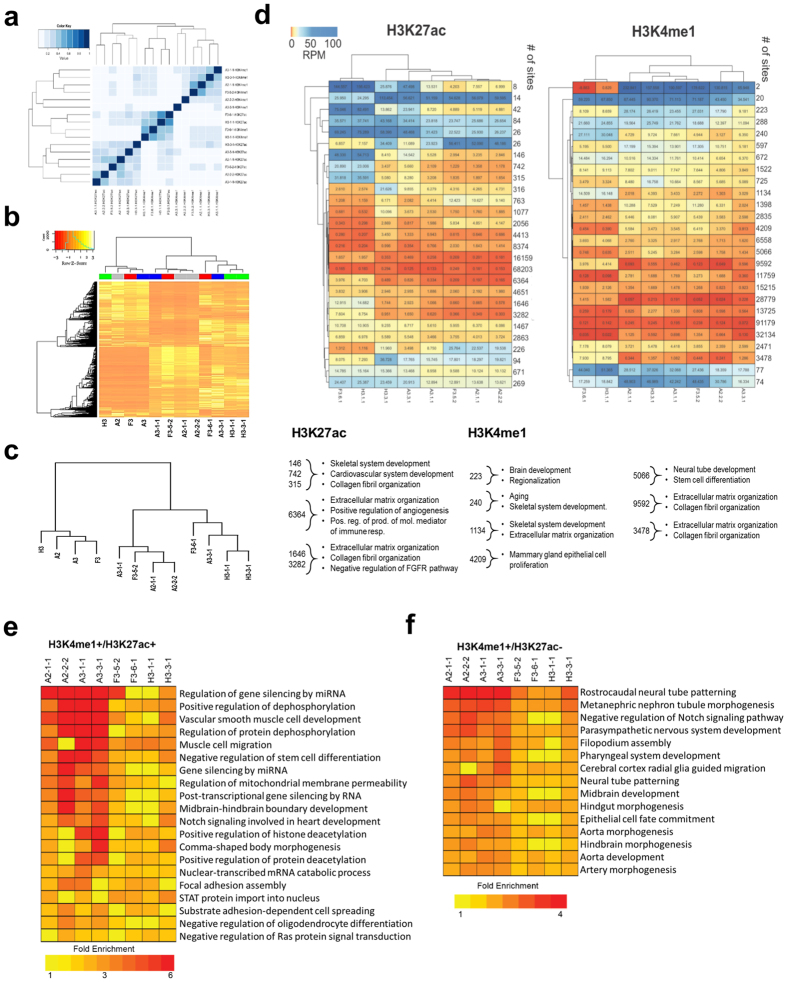
Hierarchical clustering and epigenomic profiling of ED-iPSC and initial fibroblast lines. (**a**) Jaccard analysis comparison between the ED-iPSC lines based on enriched peaks for H3K27ac and H3K4me1 ChIP-seq data. The Jaccard index is defined as the ratio of the combined length of the genomic regions within both samples (intersect) and in at least one sample (union). The A2.1.1, A2.2.2, A3.1.1, A3.3.1, F3.5.2, F3.6.1, H3.1.1, and H3.3.1 represent the eight investigated ED-iPSC cell lines. (**b**) Heatmap of hierarchical clustering using union of genes differentially expressed between ED-iPSCs and corresponding initial fibroblast cells. (**c**) Dendrogram showing the hierarchical clustering of replicate lines within self-identified ethnicity and between the ethnically diverse lines. (**d**) Comparison of ED-iPSC at the epigenomic level by clustering ChIP-seq coverage at all genomic locations for H3K27ac and H3K4me1. The number of sites for each clustered group is shown with the red to blue color gradient represents coverage value expressed as reads per million (rpm) for each sample. Significantly enriched GO-Biological process were determined by GREAT and is listed below the heatmap. Enriched GO-Biological Processes were determined for active regions defined as H3K4me1 + /H3K27ac + (**e**) and for primed regions defined as H3K4me1 + /H3K27ac- (**f**).

**Figure 8:**
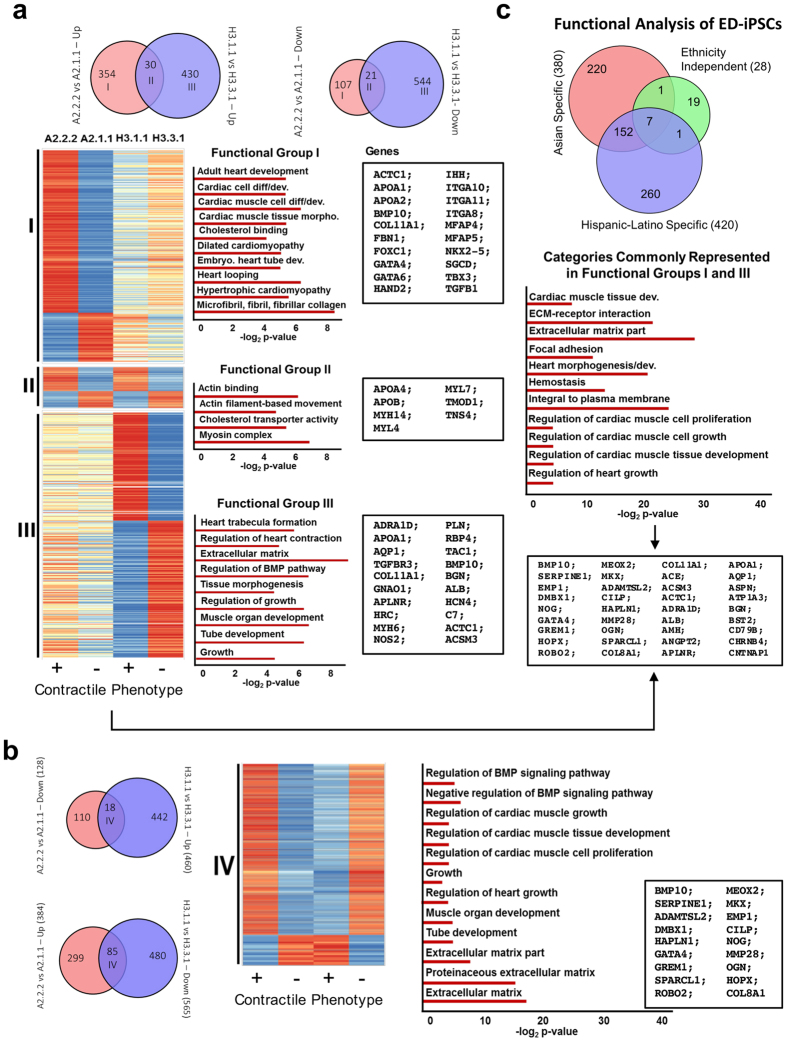
Comparative analysis of ED-iPSC contractile and non-contractile Asian and Hispanic-Latino replicate lines. (**a**) Genes concurrently up-/down-regulated between contractile and non-contractile lines in different ED-iPSCs are shown in the Venn diagrams. Heatmaps of the three different contractile-associated gene lists are shown; I: A2 dependent, II: line-independent, III: H3 dependent. Cardiac related GO categories that are uniquely overrepresented in the gene lists I, II, and III are shown next to each heatmap. Functional categories that are commonly overrepresented in A2 and H3 dependent contractile associated gene lists are also shown. Sample genes in the overrepresented categories are listed in boxes next to the category graphs. (**b**) Analysis of oppositely regulated genes between the A2 and H3 lines, with the attendant heatmap, GO categories and representative gene list. (**c**) Venn diagram comparing overlap in identified GO categories.

The importance of a 3D microarchitecture for contractility and robust Troponin-T expression can be demonstrated by over-seeding microarrays to allow a 2D cell layer to form between wells. Cells within the 2D monolayer do not stain for Troponin-T, while 3D cell aggregates present within individual wells stained for Troponin-T and exhibit banding along with a contractile phenotype (Fig. 4b). Multiple parameters were evaluated in this platform that are 3D self-assembly and Troponin-T expression (Fig. 4c–e), Troponin-T banding in extruded clusters (Fig. 4c), cardiac biomarkers (Fig. 5, NKX2.5 and GATA4), and monitoring of the contractile phenotype (Supplementary Movie 4: F3.5.2 derived cardiomyocytes). Optimization was most effective for the African American ED-iPSC line F3.5.2 but had limited effect for the Hispanic-Latino line H3.3.1 that displays diffuse Troponin-T by ICC (Fig. 3b). The differentiation efficacies of each experimentally evaluated line in this study are summarized in Table 2 and Fig. 6. To further understand the functional cardiomyocyte outcomes observed, we applied comparative bioinformatics of transcriptomes for the replicate lines from Asian or Hispanic-Latino ED-iPSCs (Figs 7 and 8).

### ED-iPSC lines reveal altered transcriptomes and gene expression profiles consistent with reprogramming

Jaccard analysis comparison between the ED-iPSC lines (Fig. 7a) based on enriched peaks for H3K27ac and H3K4me1 and analysis of differentially expressed genes allowing hierarchical clustering of ED-iPSCs versus parental fibroblasts (Fig. 7b) was done. The ED-iPSC lines are distinct from their initial fibroblasts with dramatically remodeled transcriptomes, suggesting complete reprogramming into an embryonic-like pluripotent phenotype. Raw RNA-Seq average read count and percent of high quality bases (bases with a quality score > 25) per sample were 36.85 M and 97.39%, respectively. After trimming and filtering there were 36.73 M reads and 98.12% high quality bases on average per sample (see Supplementary Fig. 2-RNA-Seq average read count and quality of ED-iPSCs). For each of the initial fibroblast cell lines labeled A2 (Asian), A3 (Asian), F3 (African American), and H3 (Hispanic-Latino), corresponding ED-iPSC lines were generated in biological replicates, labeled (A2.1.1, A2.2.2), (A3.1.1, A3.3.1), (F3.5.2, F3.6.1), and (H3.1.1, H3.3.1).

RNA-Seq analysis generated expression data for 45,989 transcripts. The lowest 2% of the expression values, in all 12 samples (i.e. 2^nd^ percentile of 12 × 45,989 signal values), was considered as the background signal value. Genes with an expression value less than the background in all 12 samples were considered “undetected” and filtered out from the downstream analysis leaving a total of 34,439 genes. Hierarchical clustering of the samples using all 34,439 genes implies that the ED-iPSCs cluster together and are separate from parental fibroblasts (Fig. 7c). Two clusters of ED-iPSCs are present. ED-iPSC replicate lines, derived from the same parental fibroblast, Asian A2.1.1/A2.2.2 and Hispanic-Latino H3.1.1/H3.3.1 fall within the same cluster group. In contrast, the African American ED-iPSC lines F3.5.2/F3.6.1 or Asian lines A3.1.1/A3.3.1 are split between the two clusters. Based on our comprehensive functional analysis (Table 1), as well as bioinformatics analysis of pluripotency markers, ED-iPSC lines within either of the two clusters were found to be pluripotent.

### Two distinct epigenetic architectures support differentiation into contractile cardiomyocytes

To further compare ED-iPSC line reprogramming at the epigenetic level, we performed ChIP-seq for histone modifications H3K4me1 and H3K27ac (Fig. 7d). H3K4me1 is found at active and primed enhancers while H3K27ac is found at active enhancers and active promoters. To further characterize active and primed chromatin states the sites containing H3K4me1 + /H3K27ac+ or H3K4me1 + /H3K27ac- were evaluated respectively (Fig. 7e,f). Epigenomic analysis of the ED-iPSC lines, as with the accompanying transcriptome analysis, reflects the stochastic nature of the reprogramming process. Even within replicate ED-iPSC pairs derived from the same fibroblast source, the ED-iPSC lines do not always cluster together, whereas different ED-iPSC lines may cluster. For example, the African American ED-iPSC line F3.6.1 and Hispanic-Latino line H3.1.1 cluster together for both modifications H3K4me1 and H3K27ac, as well as overlap of these modifications, while the replicate lines F3.5.2 and H3.3.1 fall within a separate cluster. The grouping differences for F3.6.1 and H3.1.1 are driven in part by regulatory regions associated with genes involved in extracellular matrix (ECM) organization, collagen fibril organization and cardiovascular development.

Although epigenomic clustering differences are present, this does not appear to reflect incomplete remodeling for pluripotency. ED-iPSC lines H3.1.1 as well as A2.2.2 for example, differentiated into all cell types tested in this study including contractile cardiomyocytes, and all of these ED-iPSC lines were previously validated by teratoma formation, qRT-PCR of pluripotency biomarkers, and tri-lineage commitment (Table 1 and Ref. 13). The slightly different epigenomic states, are expected to arise as a result of stochastic complexity in reprogramming^17^, and identify genes and pathways present as starting points for pluripotency and multi-lineage differentiation. Bioinformatic assessment strategies such as Pluritest^18^ are beginning to be used to compare pluripotency profiles, however, we were unable to apply Pluritest here due to the current incompatibility of our HiSeq 2500 data files with that software. Our current study provides insights into both the impact of the reprogrammed pluripotent state as well as its effect on downstream multi-lineage differentiation.

### Differential gene expression analysis and GO pathways defining contractile and non-contractile outcomes for the Asian and Hispanic-Latino ED-iPSC replicate lines reveal distinctions

Significantly differentially expressed genes were found for the following four broad comparisons: between initial fibroblasts, between ED-iPSCs, ED-iPSCs vs. initial fibroblasts, and contractile vs. non-contractile ED-iPSC biological replicates (see Supplementary Data File 1 – Differentially expressed gene comparisons). The results are summarized in Supplementary Table 1. On average, the observed difference between the ED-iPSC cell lines was less than the difference between the initial fibroblast cells. The highest difference was observed between the ED-iPSCs and the corresponding fibroblast cells, as expected. The topology of sample clustering remained unchanged when the union of differentially expressed genes (n = 8,503) between ED-iPSCs and corresponding fibroblasts was used (Fig. 7b).

Of particular interest were the replicate ED-iPSC lines in which one (A2.2.2 and H3.1.1) led to contractile cells, while the other (A2.1.1 and H3.3.1) did not. We focused on these replicate lines in order to understand the genes and corresponding functional mechanisms that could explain the observed difference in obtaining functional contractile cardiomyocytes (Fig. 8). We compared genes that were differentially expressed between contractile and non-contractile ED-iPSCs lines; A2.2.2 versus A2.1.1 and H3.1.1 versus H3.3.1. For the A2 line, there were 354 genes uniquely up-regulated and 107 genes uniquely down-regulated in the contractile ED-iPSC line compared to the non-contractile replicate (Fig. 8a). We called these 461 genes (354 + 107) “contractile-associated” genes for the A2 line. Similarly, there were 974 “contractile-associated” genes for the H3 line. There were 51 genes (30 + 21) that showed concurrent direction of expression in the contractile cells irrespective of the parental fibroblast source. We labeled these as “line-independent” contractile-associated genes. Figure 8a presents the heatmap of differentially expressed genes in the Venn diagrams, along with the cardiac related over-represented GO categories and pathways.

We identified functional categories that are uniquely or commonly overrepresented in A2 and H3 dependent contractile-associated gene lists. The functional categories that are commonly overrepresented in the A2- or H3- iPSC lines dependent gene lists are likely to show non-overlapping active regions for these categories, as the identity and the change of expression (contractile versus non-contractile) of the genes in these lists are not the same. On the other hand, categories that are uniquely associated in either the A2- or the H3-dependent iPSC lines contractile-associated gene lists show the distinct mechanisms leading to the same phenotype in the different ED-iPSC lines. Functional groups I (461 genes) and III (974 genes) are contractile-associated in the A2 or H3 lines, respectively, including up or down-regulated genes. Functional group II (51 genes) represents contractile associated genes independent of ED-iPSC line examined. We also analyzed differential expression to evaluate inversely regulated genes in comparing A2 versus H3, shown in Fig. 8b. Significantly overrepresented GO functional categories in all four gene lists can be found in Supplementary Data File 2. Finally, we generated Venn diagrams to compare overlap in identified GO pathways that included 380 from the Asian lines, 420 from the Hispanic-Latino lines, and 28 line-independent categories (Fig. 8c). Our analysis is the first to evaluate differential gene expression pathways of generated functional cardiomyocytes within reprogrammed replicate pairs comparing two distinct sources of parental fibroblasts.

## Discussion

Successful therapeutic intervention in cardiac health is influenced by population diversity. This is an area of research that stem cell biology hopes to be able to impact through the use of iPSC technology that can be applied to include a range of factors such as age, sex, ethnicity, and healthy versus disease backgrounds. Currently stem cell resources are being expanded to better reflect population diversity, including a need to increase iPSC lines generated from that ethnically diverse population^47^,^48^. An associated challenge is in defining ethnicity itself, which is a self-designated description that does not account for geographical population expansion and inter-mixing of ethnicities to various extents. Therefore, while ethnic diversity is being sought and incorporated into new iPSC line resources^13^ the current goal is to obtain findings that are more representative of the broader human population.

Here we take advantage of replicate iPSC lines that behave differently in cardiomyocyte contractility versus other multi-lineage cell differentiations to perform data-rich bioinformatic and functional analysis that informs on cardiomyocyte contractility. This study was done using replicate pairs of previously derived ethnically diverse (ED) iPSCs from fibroblasts sources obtained from apparently healthy, self-identified African American, Hispanic-Latino and Asian ethnicities, establishing a pluripotent resource evaluated by teratoma formation, qRT-PCR verified gene pluripotency profiles and multi-lineage differentiation commitment^13^. The cardiac lineage specific differences we observed in our ED-iPSC replicate lines were used in this work to understand how reprogramming can affect cardiomyocyte contractility and assist in understanding cardiomyocyte development to benefit cell therapeutic goals for cardiac health that use iPSC derived cardiomyocytes. We applied bioinformatics analysis to RNA-Seq data of replicate contractile and non-contractile ED-iPSC pairs and identify differentially expressed genes and GO pathways that may assist as predictors of contractile cardiomyocyte outcomes.

We examined three sets of ED-iPSC replicate lines for their ability to similarly differentiate into ectoderm derived pyramidal neurons, astrocytes, and RPE cells as well as endoderm derived pancreatic precursors. Following commitment to Brachyury + mesoderm, cells similarly formed smooth muscle and in the cardiac lineage expressed transcription factors NKX2.5 and GATA4. However, we observed that within and across the ED-iPSC replicate pairs there exist differences in ability to form 3D contractile aggregates in custom microarray wells and express banded (striated) Troponin-T. The replicate pairs offer a unique opportunity to understand what genes and pathways differ in generating contractile cardiomyocytes that may enable a better understanding of cardiogenesis. We performed bioinformatic analysis on the Hispanic-Latino (H) and Asian (A) replicate lines H3.1.1/H3.3.3 and A2.2.2/A2.1.1 as contractile/non-contractile pairs. Since cardiac differentiation is driven through regulation of TGF-β signaling mediated by NODAL, Activin and BMP ligands^38^,^39^,^49^,^50^ we evaluated genes in this pathway. In the A2.2.2/A2.1.1 contractile/non-contractile pair, NODAL (2.0X) and BMP10 (8X) were upregulated in ED-iPSCs for contractile cells. NODAL is also up-regulated in the H3.1.1 contractile versus H3.3.1 non-contractile ED-iPSC lines. In contrast, BMP10 (~6X) was elevated in the non-contractile H3.3.1 ED-iPSC line versus beating H3.1.1 line. BMP10 is implicated in trabeculation, or 3D remodeling, of the embryonic heart and early cardiomyocyte growth and survival but its levels are not an effective predictor of cardiomyocyte contractility across the four lines evaluated. The HAND1 transcription factor is important for morphogenic changes of cardiac looping, chamber septation and ventricular development and its mis-regulation is implicated in cardiomyopathies^51^. HAND1 is elevated in contractile A2.2.2 (~2.6X) and H3.1.1 (~2X) ED-iPSC lines versus non-contractile A2.1.1, or H3.1.1 lines. Since its expression helps to maintain the differentiating cardiac precursor state, we predict that it must be later down-regulated to drive terminal cardiomyocyte differentiation. MYL4 which encodes the Myosin Light Chain 4 protein is present in embryonic muscle and regulates muscle contractions through cyclic intracellular Ca^2+^ dependent interactions with actin-rich thin filaments. MYL4 is upregulated in both contractile lines H3.1.1 (~6X) and A2.2.2 (~5X) as compared to the non-contractile H3.3.1 and A2.1.1 lines. In addition to NODAL, HAND1, and MYL4, other genes that were similarly up-regulated in contractile versus non-contractile lines include ACTC1, COL11 A1, APOA4, APOB, and MYL7, MYH14, and GUCY1A3 and downregulation of CNTN4. Inversely regulated genes that here were not good predictors of cardiomyocyte contractile outcomes included BMP10, SERPINE1, GATA4, COL8A1, and CILP amongst others.

The gene ontology, GO, functional categories represented in each ED-iPSC contractile/non-contractile pair, shown in Fig. 7, reveal different emphasis. For example, *Group I* Asian A2 includes early development and differentiation, while *Group III,* Hispanic-Latino, includes later cardiac tissue development. Shared genes distinguishing contractile from non-contractile lines across these ED-iPSCs, referred to as line-independent *groups II and IV* in Fig. 7, include cellular infrastructure categories such as cytoskeletal remodeling, cell migration and transport, morphogenesis, focal adhesion, matrix remodeling and ECM composition, as well as functional myosin complex generation. The inability to readily form 3D aggregates and Troponin-T banding correlates with non-contractile activity in our experimental analysis. In this regard GO categories and genes involved in 3D tissue structure such as ECM remodeling, ECM-receptor interactions, actin binding and actin filament movement pathways were of interest. Cardiogenesis is known to have a strong requirement for cell-cell interactions and ECM remodeling, which can be enhanced by engineered devices and control of 3D culture formation^52^,^53^,^54^,^55^,^56^,^57^. A pathway implicated in mouse neonatal heart maturation^58^ is the cholesterol transporter pathway, which is upregulated in both our ED-iPSC Asian and Hispanic-Latino replicate lines. Of well-studied canonical pathways in cardiogenesis, such as cardiac cell differentiation, heart looping, and heart morphogenesis, these were upregulated in the contractile lines, while other pathways such as BMP regulation, tube development, cardiac muscle growth and cardiac tissue development categories included inversely regulated genes when comparing contractile versus non-contractile lines. The functional pathways revealed in the ED-iPSCs represent differences in staging preceding differentiation to cardiomyocytes.

Due to the complexity of understanding ethnicity and disease, an increasing number of large population studies in geographical regions have been done over the last decade that use genome wide association studies (GWAS) to identify risk factors in ethnic populations^59^ including African Americans^60^, Hispanic-Latino^61^, and Europeans and South Asians^62^. In addition, gene-disease associations include β1-adrenergic receptor polymorphism linked to β-blocker response^63^, proteasome subunit a6, PSMA6, linked to myocardial infarctions in Japanese^64^ and methylthioadenylsine phosphorylase, MTAP, and cyclin-dependent kinase inhibitor 2B, CDKN2B, linked to heart disease in Chinese Han populations^65^. Currently over 40 putative risk factors have been compiled from numerous recent studies^66^,^67^,^68^,^69^,^70^,^71^,^72^, that include the following genes: PPAP2B, ANKS1A, TCF21, ZC3HC1, ABO, CYP17A1, CNNM2, NT5C2, ZNF259, COL4A1, COL4A2, HHIPL1, CYP46A1, ADAMTS7, RALL, PEMT, RADS1, SRR, UBE2Z, SMCR3, PSRC1, CELSR2, SORT1, CDKN2B, SMARCA4, MRPS6, CYP17A1, AS3MT, APOA1, APOC3, APOA4, APOA5, CHRNA3, CHRNA5, SH2B3, ATXN2, TRAFD1, SMG6, TSR1, SGSM2, ABCC8, INS, MTAP, PSMA6, ADRB1, IDDM19, IDDM2, CTLA4, SLC41A2, MRS2, KCNJ11, CNNM1, NIPA1, CALPN10, PPARA, and CYP11B2. Of these genes, those differentially expressed between our contractile/non-contractile cells include: PEMT, CDKN2B, APOA1, in Asian lines and APOA4, IDDM19, PPARA and ABCC8 in Hispanic-Latino lines. While it is unclear yet the extent to which stem cell based transcriptome studies and bioinformatics analysis such as this one on ED-iPSC replicate lines can assist GWAS or other studies, it is encouraging to see overlap in risk factor genes. Cardiomyocyte therapies are showing promise for cardiomyocyte-relevant injuries such as in myocardial infarction^73^ and our analysis informs on pathways contributing to contractility that may be affected in iPSC reprogramming. Additionally, cardiac linked disease pathways of diabetes (Type 1 and Type 2) and cholesterol metabolism that are observed in larger GWAS studies, are also represented in GO pathways in our analysis of genes differentially expressed in contractile/non-contractile lines.

Our bioinformatics analysis of ED-iPSCs reveals differences in staging during cardiomyocyte differentiation, and different outcomes in contractility among replicate lines. However, the differences between each ED-iSPC line are still smaller than between ED-iPSC and parent fibroblast. GWAS and clinical trial studies are expected to benefit from *in vitro* models that can more readily provide genetic replicates to complement more complex phenotyping of large patient populations. Ethnically relevant *in vitro* models are needed and may be most valuable when generated in conjunction with GWAS studies, coupled with clinical drug studies. This study joins others that use cell line models for understanding development and disease, such as the use of cell line models to predict response to cancer drugs^74^ or a recent cardiomyocyte study published in *Cell*, in which the single mouse ES cell line E14 Tg (nkx2–5-EmGFP) was used to generate cardiomyocytes and generate a rich data set to provide insights into genes and pathways^75^,^76^. Smaller high quality data sets will continue to provide a framework for larger studies and to begin to establish robust *in vitro* models, particularly for human analysis in which animal models naturally differ from human development and function. In the current study of ED-iPSC lines we provide detailed bioinformatics to begin to understand replicate iPSC line variation in staging cardiomyocyte differentiation. Our generated ED-iPSC lines, RNA-Seq and ChIP-seq datasets, and bioinformatics analysis represent a new resource for continued investigation by the stem cell biomedical community.

## Materials and Methods

### hESC and ED-iPSC line maintenance, 2D/3D passaging, and microarray formation of uniform embryoid bodies

The previously derived human ED-iPSCs from our lab^13^ and the commercially available hESC line WA09 (WISC Bank, WiCell, Madison, WI) were maintained in mTeSR2/TeSR-E8 complete media (StemCell Technologies, Vancouver, Canada) on StemAdhere (StemCell Technologies, Vancouver, Canada) coated non-tissue culture treated dishes. Cells were enzymatically passaged between days 5–7 using the Gentle Cell Dissociation Agent (StemCell Technologies, Vancouver, Canada) with 10 μl/mL slow release bFGF2 (StemBeads FGF2; Stem Culture Incorporated, Rensselaer, NY) added at each mTeSR2/TeSR-E8 media change.

Generation of 200 μm uniform templated EBs (t-EBs) from chemically dissociated single cells was done in custom microarrays of polydimethylsiloxane (PDMS) as previously described^22^. t-EBs formed within five days in the grids with media changes every two days and removed by liquid expulsion with a p1000 micropipette (see also Fig. 1a).

### *In vitro* directed differentiation of hESC and ED-iPSC into astrocytes, pyramidal neurons, retinal pigment epithelial cells and pancreatic progenitors

t-EBs were used or single cells suspension of hESC WA09 or ED-iPSCs that were seeded into microarray wells in mTesR2/TeSR-E8 media with ROCK inhibitor (StemCell Technologies; Vancouver, Canada) and incubated overnight on Matrigel or Vitronectin-XF coated TC-treated dishes. On day 1 of differentiation, the maintenance media was replaced with Neural Induction Media (StemCell Technologies, Vancouver, Canada) and neural stem cells were generated per the protocol over 7–10 days. The resultant rosettes were then manually selected and replated onto Matrigel-coated dishes in Neural Induction Media, supplemented with 10 μM ROCK inhibitor overnight.

For astrocyte generation, adhered neural stem cells were incubated in Astrocyte Media (Sciencell Research Laboratories, Carlsbad, CA) at 37 °C and 5% CO2. Complete media changes were done every 3 days. Successful differentiation was confirmed by immunocytology to identify multiple astrocyte specific biomarkers. By day 7 (total day 17 in the protocol) in Astrocyte Media, the bulk of cells were GFAP and CD44 positive.

To generate neurons we followed a protocol for pyramidal neuron generation, essentially as described^24^,^25^,^26^, starting with templated 200 micron EBs plated onto either Matrigel or Vitronectin-XF coated dishes. Immunocytology on multiple neuronal biomarkers was performed at critical media transition steps during the differentiation protocol.

For retinal pigment epithelial (RPE) cell generation, we used an adapted protocol^27^,^32^, starting with pluripotent cells at 80–95% confluency. mRPE media (DMEM + 20% KOSR, 1x p/s, 1x NEAA, 1x GlutaMAX, 100 μM β-Mercaptoethanol) supplemented with Nicotimamide (NIC; 10 mM) and Activin-A (140 ng/ml; first 14 days) and Retinoic Acid (250 nM) was changed every other day until characteristic RPE cell morphology was achieved, usually between days 21 and 55. Once the RPE sheet was formed, media was switched back to mRPE + NIC and changed every 3 days.

To generate pancreatic progenitors, t-EBs, or single cells suspension of either hESC WA09 or ED-iPSCs were seeded into Matrigel or Vitronectin-XF coated dishes. On day 1 of differentiation, we started the StemDiff endoderm differentiation kit (StemCell Technologies). At day 5, we switched to a protocol that had been shown to generate putative β-islet cells^35^,^36^,^37^ essentially as described. Immunocytochemistry was done at day 14, day 21, day 28, and day 35 to assess differentiation. See Supplementary Figure S1 for all protocol outlines.

### *In vitro* directed differentiation of ED-iPSC to cardiomyocytes and smooth muscle cells

Templated embryoid bodies (t-EBs) were generated as described above, or single cell suspensions of either hESC WA09 or ED-iPSCs as an alternate starting material for differentiation. Both were seeded into Matrigel or Vitronectin-XF coated plates overnight, prior to starting the differentiation protocol. To initiate differentiation, we used a validated protocol^42^,^43^ essentially as described. Immunocytochemistry was performed at day 14, day 21, and day 28 to assess expression of the cardiac-specific biomarker Troponin-T. We additionally stained for the cardiac markers Nkx2.5 and GATA4 under further optimized conditions.

Generation of smooth muscle cells was done by protocol optimized from literature^44^,^45^, essentially as described. Cells were stained for alpha smooth muscle actin (α-SMA) at days 10, 14, 21, 28, and 35 to verify differentiation. See Supplementary Fig. S1 for all protocol outlines.

### Optimized cardiomyocyte differentiation protocol for ED-iPSCs lines reveals a strict Gsk3 inhibition requirement

We hypothesized that a key step in our cardiac differentiation is the initial Gsk3 inhibition (CHIR99021), as has been reported previously^42^,^43^. The A2.2.2 and H3.1.1 ED-iPSC lines can generate banded Troponin-T at 12uM GSK3 inhibitor (CHIR99021) incubation for 24 hours. By contrast, the F3.5.2 line exhibits massive cell death within 24 hours in the differentiation culture at those conditions. Cell death is greatly reduced when the GSK3 inhibitor concentration is optimized for both concentration and exposure time. Based on bioinformatics data on the A2.2.2, H3.3.1 and F3.5.2 ED-iPSC lines, the GSK3 inhibitor concentration was dropped to 6 μM, and the incubation time was further experimentally adjusted down to 6 hours, which generated banded Troponin-T + cells and spontaneously contracting areas, as well as induce contractility in LTA-PDMS generated 3D cultures (Supplementary Movies 4).

### Microscopy and immunocytology

Phase images were obtained on a Nikon 80i epifluorescence microscope with a PLAN 10 × 0.30 NA DL objective and a cooled QICam CCD camera. Fluorescent images were obtained on a Leica SP5 Laser Scanning Confocal Microscope with HC PL FLUOTAR 10 × 0.30 NA or HCX PL APO CS 20X. 70 NA objectives and on a Zeiss AxioObserver Z1 Inverted Microscope with Colibri LED illumination, 100X oil 1.45 NA PlanFLUAR or 63X Plan-Apochromat 1.4 NA oil DIC objectives, and Hamamatsu ORCA ER CCD camera using Zeiss Axiovision Rel 4.8 acquisition software. Images were compiled using Adobe Photoshop (Adobe Systems Inc., San Jose, CA) and Microsoft PowerPoint (Microsoft Corp., Redmond, WA) software. Immunocytology was done following modified fixation in 4% formaldehyde followed with a 0.5% PBST incubation overnight at 4 °C and then by a gentle wash in HBSS (Life Technologies, Grand Island, NY). Nonspecific binding was blocked by 3 × 10 minute incubations in 1% BSA-HBSS, followed by an additional HBSS wash.

Pluripotency primary antibodies used (1:1000 each) were anti-Oct4A C-10 (Santa Cruz Biotechnology, Dallas, TX). Specific cell type differentiation antibodies (1:1000 each) were used. For astrocyte differentiation, we used A2B5, CD44, GFAP, Vimentin and Nestin markers. Pyramidal neuron differentiation was screened with A2B5, MAP2, VGlut1, and Tuj1 markers. Retinal pigment epithelium (RPE) differentiation was evaluated by phase microscopy, and with the biomarkers Occludin and ZO1, which stain for epithelial tight junctions. Pancreatic differentiation was evaluated with PDX1 and Pax6 markers. Cardiomyocyte generation was stained with the Troponin-T, NKX2.5 and the GATA4 markers. Smooth muscle cells were stained for the α-smooth muscle actin marker. Secondary antibodies used (1:1000) were AlexaFluor 488 and AlexaFluor 594 (A-11001, A-11037, Invitrogen, Carlsbad, CA) and were used with Hoechst in HBSS at 4 °C overnight, followed with a final 1 hour HBSS wash at 4 °C. Mounting was then performed in ProLong Diamond (Life Technologies, Grand Island NY) at 20 °C overnight in the dark before imaging or storing in the dark at 4 °C.

### Nucleic acid preparation for RNA-Seq and ChIP-seq

Total RNA isolation for transcriptome analysis of the eight ED-iPSC lines and the four initial fibroblast cell lines were done using the Ambion PureLink Mini RNA isolation kit (Life Technologies, Grand Island, NY). Total RNA integrity and library quality control was validated using the Agilent Technologies 2100 Bioanalyzer. TruSeq RNA Sample Preparation v2 low sample (LS) protocol (Illumina, San Diego, CA) was used for mRNA preparation, library construction, and purification. Constructed RNA-Seq libraries were purified with Agencourt AMPure XP beads and quantified using the Quant-iT PicoGreen ds DNA Assay Kit (Invitrogen, Carlsbad, CA) and the KAPA Library Quantification Kit (Kapa Biosystems, Wilmington, MA) using qPCR. Libraries were normalized based on qPCR values and pooled using the TruSeq SR Cluster Kit v3-cBot-HS (Illumina, San Diego, CA). Pooled samples were four-plexed and sequenced with the HiSeq 2500 v3 sequencer (Illumina, San Diego, CA) producing 30 to 50 million single–end 50 bp reads.

Chromatin extractions from ED-iPSC cell lines were done using the Chromatin Shearing Optimization Kit - Low SDS (Diagenode, Denville, NJ) and sonicated in the Bioruptor Plus (Diagenode, Denville, NJ). Reactions were carried out with the SX-8G IP-Star^®^ Compact System (Diagenode, Denville, NJ) using the CHIP_16_IPURE_100_I protocol and purified with the MinElute PCR purification kit (QIAGEN, Valencia, CA). ChIP DNA was quantified using the Quant-iT PicoGreen ds DNA Assay Kit (Invitrogen, Carlsbad, CA) and 0.8 to 5.7 μl were used for library construction with the ThruPLEX™-FD Prep Kit (Rubicon Genomics, Ann Arbor, MI). Prepared ChIP-seq libraries were quantified with the Quant-iT PicoGreen ds DNA Assay Kit (Invitrogen, Carlsbad, CA) and the KAPA Library Quantification Kit (Kapa Biosystems, Wilmington, MA) using qPCR. Library quality control was performed with the Agilent Technologies 2100 Bioanalyzer and normalized based on qPCR, then pooled using the TruSeq SR Cluster Kit v3-cBot-HS (Illumina, San Diego, CA). Pooled samples and reference inputs were four-plexed and sequenced with the HiSeq 2500 v3 sequencer (Illumina, San Diego, CA) producing 20 to 56 million single–end 50 bp reads.

### RNA-Seq analysis

Raw reads were analyzed with FASTQC (v. 0.11.3) for quality control (Supplementary Fig. S2)^77^. Overrepresented (e.g. adapter and similar technical) sequences remaining in the raw reads were assessed and subsequently removed using the fastq-mcf module (v 1.04) under the ea-utils suite^78^. Low quality base threshold was set at^25^. Following technical sequence and low quality base removal using fastq-mcf, reads that were shorter than 20 bp were filtered out. Transcript quantification was done using the GRCh38 reference genome by RSEM (v. 1.2.22) with default parameters, which utilizes Bowtie2^79^ for read alignment^80^. RSEM uses Expectation Maximization (EM) to calculate the Maximum Likelihood (ML) estimates of expression^81^. Differential expression analysis was done using EBSeq (v. 1.10), which uses an empirical Bayes model^82^. Expression levels were subject to median normalization and genes with a fold change greater than 2 and a posterior probability of differential expression > = 0.95 were chosen (a target false discovery rate of 5%) were considered significantly differentially expressed.

For each comparison, EBSeq model convergence was manually verified, clustering of samples and/or genes was done using the Unweighted Pair Group Method with Arithmetic-mean method and Pearson’s correlation as the distance measure^83^. The Database for Annotation, Visualization and Integrated Discovery (DAVID) v6.7^84^ was used for functional analysis of the gene lists interrogating Biological Process (BP), Molecular Function (MF), and Cellular Component (CC) Gene Ontology (GO) categories^85^ and Kyoto Encyclopedia of Genes and Genomes (KEGG) pathways^86^. Biologically relevant categories that are over-represented in the gene set and therefore may be of further interest were assessed using the Expression Analysis Systematic Explorer (EASE) score in the DAVID tool. We picked GO categories that have EASE scores of 0.05 or lower as significantly over-represented.

### ChIP-seq analysis

Genomic sites enriched for the H3K27ac and H3K4me1 post-translational histone tail modifications common to one or more of the eight investigated ED-iPSC cell lines, were isolated and merged with a 200 bp window using Bedtools v2.17.0^28^,^87^, providing with a total number of 124 297 and 233 972 intervals respectively. Coverage was estimated for each of these intervals using Bedtools v2.17.0 and normalized using SAMtools v1.1^29^. Final standardized coverage values for all samples and enriched genomic sites were plotted as a heatmap with the R package Pheatmap version 0.7.7 (http://www2.uaem.mx/r-mirror/web/packages/pheatmap/index.html) for the standardized H3K27ac and H3K4me1 data respectively. GO biological processes related to differentially marked genomic sites were identified using GREAT^30^.

## Additional Information

**How to cite this article**: Tomov, M. L. *et al*. Distinct and Shared Determinants of Cardiomyocyte Contractility in Multi-Lineage Competent Ethnically Diverse Human iPSCs. *Sci. Rep.* **6**, 37637; doi: 10.1038/srep37637 (2016).

**Publisher's note:** Springer Nature remains neutral with regard to jurisdictional claims in published maps and institutional affiliations.

## Supplementary Material

Supplementary Information

Supplementary Movie S1

Supplementary Movie S2

Supplementary Movie S3

Supplementary Movie S4

Supplementary Dataset 1

Supplementary Dataset 2

## Figures and Tables

**Table 1:** ED-iPSC Summary of Analysis Performed.

iPSC Line	Fibroblast Source^a^	Fibroblast Pathology^a^	iPSC Karyotype^b^	iPSC Pluripotency^b^	iPSC Teratoma^b^,^c^	iPSC Multi-Lineage Diff.^d^,^e^	iPSC RNA-Seq^d^	iPSC ChIP-seq^d^
Details	As reported	As reported	Tested for abnormalities	ICC Tested PCR Tested	Germ layer expression	*In vitro* targeted differentiation	HighSeq 2500 v3 Four-plexed	HighSeq 2500 v3 Four-plexed
A2.1.1	Coriell Institue	Apparently healthy	Normal XY	ICC - Oct4A, Nanog, SSEA-4; RT-PCR – Oct4, Sox2, Nanog, Rex1	Ectoderm + Endoderm + Mesoderm+	Pancreatic; Neuronal; Astrocyte; Smooth muscle; Cardiac muscle	30 to 50 million single-end 50 bp reads	H3K27ac; H3K4me1
A2.2.2	Coriell Institue	Apparently healthy	Normal XY	ICC - Oct4A, Nanog, SSEA-4; RT-PCR – Oct4, Sox2, Nanog, Rex1	Ectoderm + Endoderm + Mesoderm+	Pancreatic; Neuronal; Astrocyte; Smooth muscle; Cardiac muscle	30 to 50 million single-end 50 bp reads	H3K27ac; H3K4me1
F3.5.2	Coriell Institue	Apparently healthy	Normal XY	ICC - Oct4A, Nanog, SSEA-4; RT-PCR – Oct4, Sox2, Nanog, Rex1	Ectoderm + Endoderm + Mesoderm+	Pancreatic; Neuronal; Astrocyte; Smooth muscle; Cardiac muscle	30 to 50 million single-end 50 bp reads	H3K27ac; H3K4me1
F3.6.1	Coriell Institue	Apparently healthy	Normal XY	ICC - Oct4A, Nanog, SSEA-4; RT-PCR – Oct4, Sox2, Nanog, Rex1	Ectoderm + Endoderm + Mesoderm+	Pancreatic; Neuronal; Astrocyte; Smooth muscle; Cardiac muscle	30 to 50 million single-end 50 bp reads	H3K27ac; H3K4me1
H3.1.1	Coriell Institue	Apparently healthy	Normal XY	ICC - Oct4A, Nanog, SSEA-4; RT-PCR – Oct4, Sox2, Nanog, Rex1	Ectoderm + Endoderm + Mesoderm+	Pancreatic; Neuronal; Astrocyte; Smooth muscle; Cardiac muscle	30 to 50 million single-end 50 bp reads	H3K27ac; H3K4me1
H3.3.1	Coriell Institue	Apparently healthy	Normal XY	ICC - Oct4A, Nanog, SSEA-4; RT-PCR – Oct4, Sox2, Nanog, Rex1	Ectoderm + Endoderm + Mesoderm+	Pancreatic; Neuronal; Astrocyte; Smooth muscle; Cardiac muscle	30 to 50 million single-end 50 bp reads	H3K27ac; H3K4me1
H3.5.2	Coriell Institue	Apparently healthy	Normal XY	ICC - Oct4A, Nanog, SSEA-4; RT-PCR – Oct4, Sox2, Nanog, Rex1	Ectoderm + Endoderm + Mesoderm+	Pancreatic; Neuronal; Astrocyte; Smooth muscle; Cardiac muscle	30 to 50 million single-end 50 bp reads	H3K27ac; H3K4me1

^a^Information available from the Coriell Institute.

^b^Analysis described in Chang *et al*. (ref. 13).

^c^Histology included in Chang *et al*.

^d^Current study.

^e^See also Table 2 in current study.

**Table 2:** Targeted Differentiation Efficacy (N > 3; 200 cells per line per experiment).

iPSC Line	Pancreatic Diff.	Effic. (%)	St. D. (%)	Pyramidal Neuron Diff.	Effic. (%)	St. D. (%)	Astrocyte Diff.	Effic. (%)	St. D. (%)	Smooth Muscle Diff.	Effic. (%)	St. D. (%)
A2.1.1	Figs 1 & 2c	97.50	2.50	Figs 1 & 2b	45.00	1.44	Figs 1 & 2a	98.33	1.44	Fig. 3	83.33	6.29
A2.2.2	Figs 1 & 2c	96.67	1.44	Figs 1 & 2b	40.83	1.44	Figs 1 & 2a	97.50	1.44	Fig. 3	81.67	8.04
F3.5.2	Figs 1 & 2c	96.67	1.44	Figs 1 & 2b	41.67	1.44	Figs 1 & 2a	99.17	1.44	Fig. 3	82.50	4.33
F3.6.1	Figs 1 & 2c	87.50	2.50	Figs 1 & 2b	42.50	2.50	Figs 1 & 2a	99.17	1.44	Fig. 3	75.00	3.82
H3.1.1	Figs 1 & 2c	97.50	2.50	Figs 1 & 2b	41.67	5.20	Figs 1 & 2a	96.67	1.44	Fig. 3	84.17	3.82
H3.3.1	Figs 1 & 2c	96.67	1.44	Figs 1 & 2b	42.50	2.50	Figs 1 & 2a	97.50	2.50	Fig. 3	80.83	3.82
H3.5.2	Figs 1 & 2c	95.83	2.89	Figs 1 & 2b	44.17	2.89	Figs 1 & 2a	99.17	1.44	Fig. 3	87.50	2.50
WA09	Figs 1 & 2c	97.50	2.50	Figs 1 & 2b	40.83	3.82	Figs 1 & 2a	99.17	1.44	Fig. 3	85.83	5.20

Note: F3.6.1 line shows small, but statistically significant differences in pancreatic and smooth muscle differentiation efficiency.
