# Supplementary material for: Distinct and Shared Determinants of Cardiomyocyte Contractility in Multi-Lineage Competent Ethnically Diverse Human iPSCs
Source: Sci Rep. 2016 Dec 5;6:37637. doi: 10.1038/srep37637 (PMC5137163; doi:10.1038/srep37637)

Distinct and Shared Determinants of Cardiomyocyte Contractility in Multi-Lineage Competent Ethnically Diverse Human iPSCs

Martin L. Tomov, Zachary T. Olmsted, Haluk Dogan, Eda Gongorurler,
Maria Tsompana, Hasan H. Otu, Michael Buck, Eun-Ah Chang, Jose Cibelli and Janet L. Paluh

Supplementary Figures Legends

Supplementary Table 1. List of differentially expressed genes. Row ***i***, column ***j*** represents genes up-regulated in sample "***j***" compared to sample "***i***".

Supplementary Figure S1. ED-iPSC lines differentiation protocols.

Detailed protocol outlines used to generate **(a)** astrocytes, **(b)** pyramidal neurons, **(c)** retinal pigment epithelial (RPE) cells, **(d)** pancreatic progenitors, **(e)** smooth muscle cells, and **(f)** cardiomyocytes, either using the standard protocol, or the ED-iPSC optimized one.

Supplementary Figure S2. RNA-Seq average read Counts and quality of ED-iPSCs raw data.

Analysis of ED-iPSC read counts and raw output show high quality RNA-Seq data.

Supplementary Data File 1. Differentially expressed gene list.

Differentially expressed genes in fibroblast parent lines compared to ED-iPSCs, ED-iPSCs vs. initial fibroblasts, and contractile vs. non-contractile ED-iPSC biological replicates.

Supplementary Data File 2. GO and pathway lists.

Full list of differentially expressed GO terms and pathways in the ED-iPSC replicate lines.

Supplementary Movie 1: Mixed cardiomyocyte culture with contractile and non-contractile regions.

H9 hESC derived cardiomyocytes grown on Matrigel coated dish show random non-contractile 2D regions and contractile 3D self-assembled regions within the same culture by day 15. Movie is at 1X speed, phase acquisition.

Supplementary Movie 2: Multi-well array of aggregated contractile cardiomyocytes.

High density seeded H9 hESC 2D culture derived cardiomyocytes were dissociated into single cells and seeded within the microwell array. Cells form 3D contractile clusters when allowed to self-assemble within patterned microwells by day 5. Movie is at 1X speed, phase acquisition. Well diameter is 200 µm. Contractile clusters we observed in 396/400 wells in three separate experiments.

Supplementary Movie 3: Zoom-in of multi-well array of aggregated contractile cardiomyocytes.

High density seeded H9 hESC 2D culture derived cardiomyocytes were dissociated into single cells and seeded within the microwell array. The cells preferentially form 3D contractile clusters compared to inter-well areas, when allowed to self-assemble within patterned microwells by day 5. Movie is at 1X speed, phase acquisition. Well diameter is 200 µm. Contractile clusters we observed in 790/800 wells spread over three separate experiments.

Supplementary Movie 4: Aggregated cardiomyocytes contract in microwell arrays.

2D culture F3.5.2 derived cardiomyocytes were dissociated into single cells and loaded into a microwell array. Cells preferentially self-assemble into contractile 3D clusters in templated wells within 5 days. Movie is at 1X speed, phase acquisition. Well diameter is 200 μm.

Supplementary Table 1. List of differentially expressed genes. Row ***i***, column ***j*** represents genes up-regulated in sample "***j***" compared to sample "***i***".

<table>
<tr><th></th><th>A2</th><th>A3</th><th>F3</th><th>H3</th><th>A2.1.1</th><th>A2.2.2</th><th>A3.1.1</th><th>A3.3.1</th><th>F3.5.2</th><th>F3.6.1</th><th>H3.1.1</th><th>H3.3.1</th></tr>
<tr><td>A2</td><td></td><td>446</td><td>578</td><td>665</td><td>2122</td><td>2617</td><td></td><td></td><td></td><td></td><td></td><td></td></tr>
<tr><td>A3</td><td>536</td><td></td><td>204</td><td>586</td><td></td><td></td><td>2199</td><td>2584</td><td></td><td></td><td></td><td></td></tr>
<tr><td>F3</td><td>569</td><td>241</td><td></td><td>564</td><td></td><td></td><td></td><td></td><td>2174</td><td>2026</td><td></td><td></td></tr>
<tr><td>H3</td><td>892</td><td>795</td><td>793</td><td></td><td></td><td></td><td></td><td></td><td></td><td></td><td>2637</td><td>2664</td></tr>
<tr><td>A2.1.1</td><td>2431</td><td></td><td></td><td></td><td></td><td>384</td><td rowspan="2" colspan="2">282</td><td rowspan="2" colspan="2">87</td><td rowspan="2" colspan="2">2154</td></tr>
<tr><td>A2.2.2</td><td>2106</td><td></td><td></td><td></td><td>128</td><td></td></tr>
<tr><td>A3.1.1</td><td></td><td>2382</td><td></td><td></td><td rowspan="2" colspan="2">481</td><td></td><td></td><td rowspan="2" colspan="2">109</td><td rowspan="2" colspan="2">697</td></tr>
<tr><td>A3.3.1</td><td></td><td>1782</td><td></td><td></td><td></td><td></td></tr>
<tr><td>F3.5.2</td><td></td><td></td><td>2159</td><td></td><td rowspan="2" colspan="2">242</td><td rowspan="2" colspan="2">170</td><td></td><td></td><td rowspan="2" colspan="2">702</td></tr>
<tr><td>F3.6.1</td><td></td><td></td><td>1727</td><td></td><td></td><td></td></tr>
<tr><td>H3.1.1</td><td></td><td></td><td></td><td>1777</td><td rowspan="2" colspan="2">1882</td><td rowspan="2" colspan="2">377</td><td rowspan="2" colspan="2">226</td><td></td><td>565</td></tr>
<tr><td>H3.3.1</td><td></td><td></td><td></td><td>1767</td><td>460</td><td></td></tr>
</table>

Supplementary Figure S1, Tomov et al., 2016

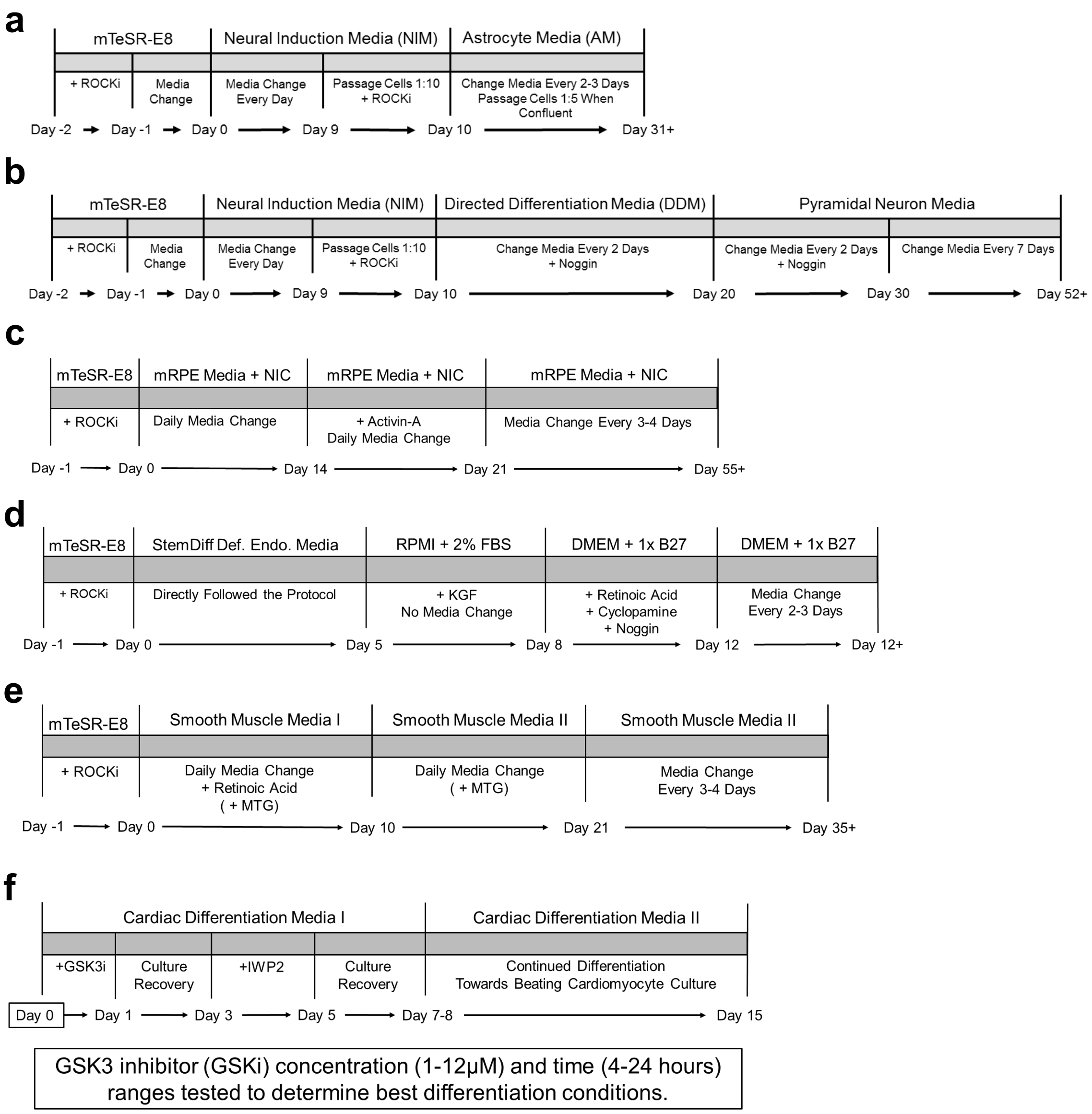

Supplementary Figure S2, Tomov et al., 2016

a

	RAW	Adapter Cleaned and Quality	RAW				Adapter Cleaned and Quality Filtered			
Internal ID	Read Number		Base Pair							
	Pair 1	Pair 1	Pair 1	Avg. Read L	Pair 1 Q >= 25	%High Quality	Pair 1	Avg. Read L	Pair 1 Q >=25	%High Quality
A2	31029390	31017103	1582498890	51	1558816472	98.503	1542960933	49.745	1526165683	98.911
A2-1-1	32958431	32797882	1680879981	51	1636397206	97.354	1625900520	49.573	1594245302	98.053
A2-2-2	40239117	40209471	2052194967	51	1997615263	97.340	1993004965	49.566	1953793458	98.033
A3	27747546	27737279	1415124846	51	1395084863	98.584	1380254250	49.762	1365977936	98.966
A3-1-1	31487964	31381523	1605886164	51	1563110855	97.336	1555319470	49.562	1524922165	98.046
A3-3-1	50236778	50199809	2562075678	51	2494815251	97.375	2488364516	49.569	2440198052	98.064
F3	22671797	22662955	1156261647	51	1139690980	98.567	1127667203	49.758	1115896687	98.956
F3-5-2	50417664	50350752	2571300864	51	2475643127	96.280	2485527946	49.364	2417730252	97.272
F3-6-1	43614053	43445144	2224316703	51	2142163924	96.307	2144590146	49.363	2086183502	97.277
H3	28837478	28249045	1470711378	51	1450673205	98.638	1289442517	45.646	1281913440	99.416
H3-1-1	39526078	39337609	2015829978	51	1938890671	96.183	1941077594	49.344	1886157677	97.171
H3-3-1	43463804	43418156	2216654004	51	2133812239	96.263	2142710414	49.351	2083912084	97.256
Average	36852508.333	36733894.000	1879477925			97.394	1809735040			98.118
StDev	9031668.717	9060773.218				0.988407284				0.787366744

b –

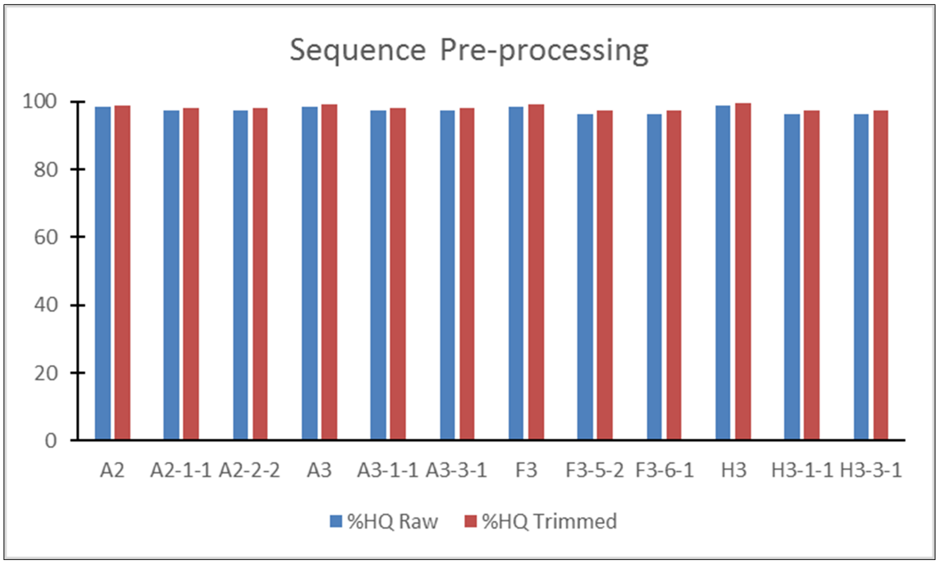

Supplement: Supplementary Information [file srep37637-s1.pdf]
